# Triumphs of Young Physic, or, Chrono-Thermal Facts

**Published:** 1847-08

**Authors:** William Turner

**Affiliations:** Late Health Commissioner of the City and County of New-York—Member of the N. Y. Medical Society—American Editor of the Principles of the Chrono-Thermal System of Medicine, &c., &c


ART. III.—*Triumphs of Young Physic, or, Chrono-thermal facts.* By William Turner Esq., A. M., M. D. Late Health commissioner of the city and county of New-York—member of the N. Y. Medical Society—American Editor of the principles of the Chrono-thermal system of Medicine, &c., &c

"Let's purge this choler without letting blood,
* * * conclude, and be agreed;
Our doctors say, this is no time to bleed."—*Richard II.*

"The day is not distant when quacks only will resort to the lancet and the leech for any disease."—*Dickson.*

New-York, Burgess, Stringer & Co; Wm. H. Graham; W. Taylor & Co; H. Long and Brother; Philadelphia, Zieber & Co; Boston, Reading & Co.

Some one has been kind enough to send us a tract with the above formidable title page, which we have given at length, not omitting the list of publishers. We should have added, that the work is an octavo of thirty-one pages, and that it may doubtless be had for the asking, without money, and without price. The chrono-thermal system, as it is called, purports to be a new discovery in pathology and therapeutics, of which a Dr. Dickson, of London, is the fortunate author. We must confess that we are not prepared to state in what the system consists except that it repudiates bloodletting, and contends for a principle of periodicity in all diseases. Suffice it to say, it is a new *system*, and, although we presume nine-tenths of the profession know but little of it, and care less, yet, if its few votaries were to be believed, we are all full of apprehension in view of the imminent danger in which legitimate medicine stands of being entirely subverted.

This Dr. Turner claims to be the American expounder of the principles of the new doctrine. Although he professes to think very meanly of the medical profession, he appears to be anxious to have his readers know that he is an M. D., and a member of the New-York Medical Society. Strange that he should regard these distinctions worthy to be attached to his name! One might suppose that to be the "*American Editor of the principles of the chrono-thermal system of Medicine*," would be honor enough, and that allusion to his nominal connection with a class which he so thoroughly despises, would, from choice, have been dispensed with! But Dr. Turner is not singular in this respect. We have often noticed, that when a person is enticed from the honorable walks of the profession, by the temptations held out by empiricism, he never relinguishes the symbolical insignia of his former connection. The profession would like to have the separation complete. Not so the reformed doctor; he clings tenaciously to that which he professes to hold in contempt and defiance. But this same Dr. Turner has held the situation of *Health Commissioner of the city and county of New-York*. We recollect it was commented on at the time, as a melancholy instance of the humiliations inflicted upon the medical profession.

We do not intend to waste our time, nor trifle with the patience of our readers, so far as to review the little pamphlet before us. It belongs to the class of publications, of which thousands are yearly disseminated over the country, designed to catch gulls, by professing to cure all diseases by newly invented, and never failing methods. It professes to give a list of remarkable cures of the various ills that flesh is heir to, and concludes with a host of testimonials to the efficacy of the system.

As a matter of curiosity we have read the work, and our attention was attracted by two letters recommending the doctrine to general adoption. The letters to which we refer, bear the signatures of gentlemen of some distinction in a sister profession—the Law. This fact arrested our attention, and gave rise to some reflections. The names of the others, being unknown to fame, we have only to presume that they have their living representatives somewhere, but whether, if we knew the individuals, we should deem their favorable testimony recommendatory, or quite the reverse, is a matter of uncertainty. Hon. Willis Hall, and Ogden P. Edwards, are, however, persons above the common order of those who are willing to become *stool pigeons*, for their neighbors to be caught and plucked. The first named gentleman was formerly the Attorney General of this State, the latter is an eminent jurist in the city of New-York. As these gentlemen have so high regard for chrono-thermalology as to lend their names as endorsers for its benefit, they will not object to our doing what little is in our power to disseminate the fact. We will do them, and the system, the justice to quote their language.

Hon. Willis Hall writes as follows:—

"I have just received your book. I need not express my opinion of the work, for you know it already. I have heretofore read parts of it with much pleasure and profit. I will merely say, what I do not recollect mentioning to you, that the intermittent nature of disease continually appears, so far as my case (palsy from being bled) is concerned. The changes backwards and forwards on different days are very considerable. I have not yet detected the law which governs it, whether it is alternate, tertian, qnartan, &c. I trust and believe the publication may do good. It will tend to open the eyes of the world."

"We had large armies in Flanders," was the quaint remark of uncle Toby, in answer to the expression of surprise by Dr.Slop, that children were even born alive before his forceps were invented! In like manner we may say, that thousands have bled, and in true Sangrado fashion, for the last half century, (not a few injudiciously as all will admit) but, strange to say, the occurrence of palsy as a result, has not before been noticed. This is a new development in the etiology of that affection, which it remained for Mr. Hall to ascertain. We will exercise a delicacy which Mr. H. appears not to esteem due to his medical advisers, and simply remark upon a statement, the absurdity of which might justly enough give occasion for ridicule, that we fear paralysis following an apoplectic seizure denotes something more serious than any effect which bleeding, however injudicious it may have been, could have produced.

The following is the concluding paragraph of Mr. Edwards' letter:—

"I suppose that the Chrono-Thermal system will be pronounced a humbug by those learned leeches who keep the gates of that great medical turnpike leading to "that bourne from which no traveller returns." But notwithstanding the opinion of dogmatic therapeutics, I must speak well of the bridge that carries me safely over, and am compelled to think that health is better than medical metaphysics, and sound joints than calomel, and that I would rather be cured irregularly than be sent to the grave *secundum artem*."

Now, as lawers, both holding, or having held, high official stations, we entertain for these gentlemen none other than sentiments of profound respect. We doubt not each is learned in the law, and that the opinion of each on a legal question would be sound and reliable. But legal knowledge is not universal knowledge. We opine there are some things, be their legal acquirements and acumen never so great, that they are but poorly qualified to decide upon. It is more than probable that medicine is one of these. The laws of Nations and Legislatures have but few relations with the laws of disease, the administration of justice is quite different from the application of the principles of therapeutics, the body politic bears but a faint analogy to the body animate. They would doubtless be ready enough to appreciate the force of this remark if a physician were to claim on the score of his medical knowledge competency to decide in matters of law. This would be deemed arogant, and a fit subject for decision. But the converse of this is not so readily recognised, by them, as equally ridiculous.

We will not pursue this train of reflection, for we can doubtless occupy our pages with matter more profitable to our readers. We should only express truths which every medical man has abundant opportunities to perceive and feel. We will only say, in conclusion, that both these gentlemen, if their lives are spared, will doubtless sooner or latter, have occasion, either in their own persons, or in the cases of friends in whom they feel a deep interest, to seek assistance from some of the "learned leeches who keep the gates of the great medical turnpike leading to that 'bourne from which no traveller returns,'" and who send their patients to the grave *seeundum artem.*

We say this with entire confidence that our prediction will be verified—a confidence not based on any acquaintance with them personally, but upon knowledge of similar instances. Thus it is, that our profession heaps coals of fire on the heads of those who have insulted and abused it—and we could desire no surer, nor better revenge.

